# Psychometric Properties of the Persian Version of the Handwriting Proficiency Screening Questionnaire in Iranian Primary School Students in Grades 2 and 3

**DOI:** 10.34172/hpp.45288

**Published:** 2026-08-21

**Authors:** Naser Havaei, Mina Ahmadi Kahjoogh, Hamid Reza Mohtaram Nejhad, Ahmad Mohammadi, Babak Kashefimehr

**Affiliations:** Department of Occupational Therapy, Faculty of Rehabilitation Sciences, Tabriz University of Medical Sciences, Tabriz, Iran

**Keywords:** Handwriting, Psychometrics, Child, Iran

## Abstract

**Introduction:**

Handwriting difficulties are prevalent among primary school children and can adversely affect academic achievement, self-confidence, and overall school participation. This study aimed to evaluate the psychometric properties of the Persian version of the Handwriting Proficiency Screening Questionnaire (HPSQ) in Iranian second- and third-grade students.

**Methods:**

This methodological study was conducted in primary schools in Tabriz, Iran. A total of 203 typically developing students (95 second-graders, 108 third-graders; 103 boys) aged 8–10 years participated. The content and construct validity of the HPSQ were examined. To assess reliability, the internal consistency and test-retest reliability of the questionnaire were evaluated.

**Results:**

CVR and CVI were 0.8-1 and 0.9-1 respectively. The results of the discriminant validity analysis showed that the questionnaire has the ability to distinguish between the handwriting skills of second and third grade students. Principal axis factoring indicated that the items of the Persian version of HPSQ were loaded into two components. Confirmatory factor analysis also confirmed the results of EFA. The findings suggested excellent internal consistency (α=0.93). Test-retest of the HPSQ ranged from 0.89- 0.96.

**Conclusion:**

The Persian HPSQ is a valid and reliable screening tool for handwriting difficulties in Iranian primary school students in grades 2 and 3. While some fit indices suggest potential for improvement, the overall findings support its use as a screening tool for handwriting difficulties in Iranian primary school students.

## Introduction

 Handwriting as a complex skill consists of different components requiring the integration of sensory-motor and cognitive systems.^1,2^ People at all stage B s of life require legible handwriting to communicate and express their ideas.^1,3^ Children need this skill to succeed in their school activities.^1^ Students with illegible handwriting experience problems with learning and mathematics, as well as in daily affairs, with approximately two-thirds of school assignments requiring handwriting skills.^3,4^ Several cognitive, motor and linguistic factors can affect children’s handwriting. One study showed that visual-motor integration is a key determinant of handwriting proficiency in children of different ages.^5^ Poor handwriting is prevalent among children diagnosed with autism spectrum disorders, developmental coordination disorders, and learning disorders.^6-8^

 Improving handwriting is a primary goal of occupational therapy interventions for children.^1^ Teachers can guide students with handwriting problems and refer them for occupational therapy interventions. Writing speed and legibility are two critical aspects often considered in evaluation.^1,9,10^ Children with handwriting problems typically write slower than their peers and struggle with organizing and copying letters and words.^11^

 Given the high prevalence of handwriting impairments in children,^12^ valid and reliable tools are essential for measurement. The Handwriting Proficiency Screening Questionnaire (HPSQ) is a tool that uses teachers’ opinions to measure students’ handwriting problems.^13^ The HPSQ has been translated into several languages ^3,4,14^ and is easy to use, requiring little time to complete. In addition to assessing speed and legibility, it examines emotional and physical aspects of writing.^15^ The purpose of this study was to translate the HPSQ into Persian and examine its psychometric properties in Iranian school students.

## Methods

 This methodological and cross-sectional study was conducted in two phases: translation and psychometric evaluation.

###  Translation Phase

 The translation process followed a forward-and-backward procedure. After obtaining permission from the developer, two occupational therapists experienced in school-based practice and a certified translator translated the questionnaire into Persian. The final version was agreed upon in a meeting with the translators. After completing the forward Persian translation, the questionnaire was translated again into English and was approved by the developer. The backward translation was done by three certified translators who were fluent in English.

###  Psychometric Properties Phase 

 Once the final version of the HPSQ was determined, the content and construct validity (including discriminant ability, exploratory factor analysis [EFA], confirmatory factor analysis [CFA]) were examined. Reliability was assessed through internal consistency and test-retest reliability. The Persian version of the HPSQ is provided in the Appendix.

###  Participants 

 Two hundred three typically developing second-grade (n = 95) and third-grade (n = 108) students participated (boy = 103). The inclusion criteria were: children in grades 2 or 3, aged 8 to 10 years, without documented physical and mental dysfunctions. Elementary school teachers with over ten years of experience were selected to complete the questionnaire. Participants were excluded if they had received special education or were unwilling to participate. Schools were selected from different areas of Tabriz, Iran, and students chosen via simple random sampling. The sample size was determined based on established guidelines for factor analysis. A sample size of 203 participants (approximately 20 participants per item) meets the minimum recommended ratio of 10:1 for factor analysis.^16^

###  Procedure 

 All participants were informed about the study purpose and provided written informed consent. Students were selected using simple random sampling from primary schools (n = 203). The Persian language teacher for each student filled out the Persian version of the HPSQ and expressed their opinions about the handwriting status. To assess test-retest reliability, teachers for 43 students completed the questionnaire again after a two-week interval.

###  Instrument

 The HPSQ was developed by Rosenblum (2008) to identify handwriting problems in school-aged children.^13^ This questionnaire contains ten items: 1. Is the child’s writing unreadable? 2. Is the child unsuccessful in reading his/her own handwriting? 3. Does the child not have enough time to copy tasks from the blackboard? 4. Does the child often erase while writing? 5. Does the child often feel he/she does not want to write? 6. Does the child not do his/her homework? 7. Does the child complain about pain while writing? 8. Does the child tire while writing? 9. Does the child need to look at the page/blackboard often when copying? 10. Is the child not satisfied with his/her handwriting?

 A 5-point Likert-scale is used for scoring, such that 0 refers to never and 4 refers to always; higher scores indicate poorer performance.

###  Data Analysis

 Descriptive statistics summarized participant characteristics. The Content Validity Index (CVI) and the Content Validity Ratio (CVR) were determined. A panel of experts (including 6 pediatric occupational therapists, 3 primary school teachers, 1 physiotherapist and 2 speech and language pathologists) scored the CVR on a three-point Likert scale. The CVI was determined using a four-point Likert scale for item simplicity, specificity, and clarity.

 Discriminant ability was checked by comparing scores of second and third-grade students using the Mann-Whitney U test.

 Principal axis factoring with promax rotation was used to examine the nature of the interrelationship between the Persian version of the HPSQ measurements and the group-correlated measurements. The appropriateness of the factor analysis model and the sampling adequacy were assessed using Bartlett’s test of sphericity and the Kaiser-Meyer-Olkin test, respectively. The number of factors was determined using eigenvalues greater than 1 and a scree plot. CFA was performed using Lisrel 8.80 to test the two-dimensional structure identified by EFA.

 Cronbach’s alpha was used to assess internal consistency. Cronbach’s alpha values were interpreted as follows: > 0.9, 0.8-0.9, 0.7-0.8, 0.6-07, 0.5-0.6, and < 0.5 are considered to be excellent, good, acceptable, questionable, poor, and unacceptable, respectively.^17^ Test-retest reliability was assessed using the Intraclass Correlation Coefficient (ICC) with a two-week interval (n = 43).

## Results

 A total of 203 typically developing students (95 second-graders, 108 third-graders; 103 boys, 99 girls) aged 8–10 years participated in this study. The mean age of second-grade students was 8.07 ± 0.11 years, and the mean age of third-grade students was 9.03 ± 0.115 years. Regarding hand dominancy, 181 students were right-handed and 22 students were left-handed.

 The CVR and CVI, used to assess content validity were 0.8-1 and 0.9-1 respectively, which are acceptable (Table 1).

**Table 1:** CVR and CVI Values for HPSQ (n = 12)

**Item**	**CVR**	**CVI (relationship)**	**CVI (clarity)**	**CVI (simplicity)**
1	1	1	1	1
2	0.8	1	0.9	1
3	1	1	1	0.9
4	0.8	1	1	1
5	0.8	0.9	1	0.9
6	0.8	1	1	1
7	0.8	0.9	0.9	1
8	1	1	1	1
9	1	1	1	0.9
10	0.8	1	0.9	1

CVR: Content Validity Ratio, CVI: Content Validity Index

 For discriminant ability (Table 2), the handwriting scores of the second-grade students were compared with those of the third-grade students, and the results showed that the HPSQ can distinguish between the handwriting of these two grades (*P* < 0.05). Effect sizes (Cohen’s d) were also calculated to assess the magnitude of differences between grades. As shown in Table 2, the effect sizes ranged from 0.4 to 0.98, with 6 items demonstrating medium to large effects (d ≥ 0.50). This indicates that the observed differences between grade 2 and grade 3 students are not only statistically significant but also practically meaningful.

**Table 2:** Comparison of the Mean and Standard Deviation of Variables Between Students in Grades Two and Three (Discriminant Validity) – Nonparametric Mann-Whitney Test (n = 203, grade 2 = 95/ grade 3 = 108)

**Item**	**Grade**	**Mean±SD**	***P*** **-value**	**Cohen's d**
1	2 3	1.2 ± 0.932 0.79 ± 1.142	0.002	0.4
2	2 3	1.18 ± 1.051 0.57 ± 0.989	0.000	0.59
3	2 3	1.53 ± 1.099 0.79 ± 1.031	0.000	0.7
4	2 3	1.42 ± 1.009 0.93 ± 0.977	0.003	0.49
5	2 3	1.14 ± 0.943 0.44 ± 0.775	0.000	0.81
6	2 3	1.06 ± 0.875 0.32 ± 0.619	0.000	0.98
7	2 3	0.95 ± 0.885 0.33 ± 0.811	0.000	0.73
8	2 3	1.09 ± 0.872 0.73 ± 0.977	0.005	0.4
9	2 3	1.47 ± 1.056 1.04 ± 1.202	0.011	0.4
10	2 3	1.06 ± 0.875 0.64 ± 1.111	0.000	0.42
Total	2 3	12.1 ± 8.06 6.58 ± 7.07	0.000	0.73

SD: Standard Deviation

 Principal axis factoring with promax rotation showed that the items in the HPSQ were loaded into two components (Table 3). The promax rotation revealed a moderate correlation between the two extracted components (r = 0.7), justifying the use of an oblique rotation.

**Table 3:** Factor Loading of the Measurements of the Persian version of HPSQ in the Principal Axis Factoring and Rotated Using Promax (EFA) (n = 203)

**Item**	**Component 1**	**Component 2**
1		0.93
2		0.88
3	0.756	
4	0.805	
5	0.663	
6	0.706	
7	0.647	
8	0.842	
9	0.89	
10		0.796

 The two factors together accounted for 73.96% of the total variance. The first factor had an eigenvalue of 6.35 and explained 63.39% of the variance. The second factor had an eigenvalue of 1.04 and explained 10.44% of the variance. The scree plot (Figure 1) showed a clear inflection point after the second factor, supporting the retention of two components. No significant cross-loadings were observed, as all items loaded cleanly onto their respective factors.

**Figure 1:**
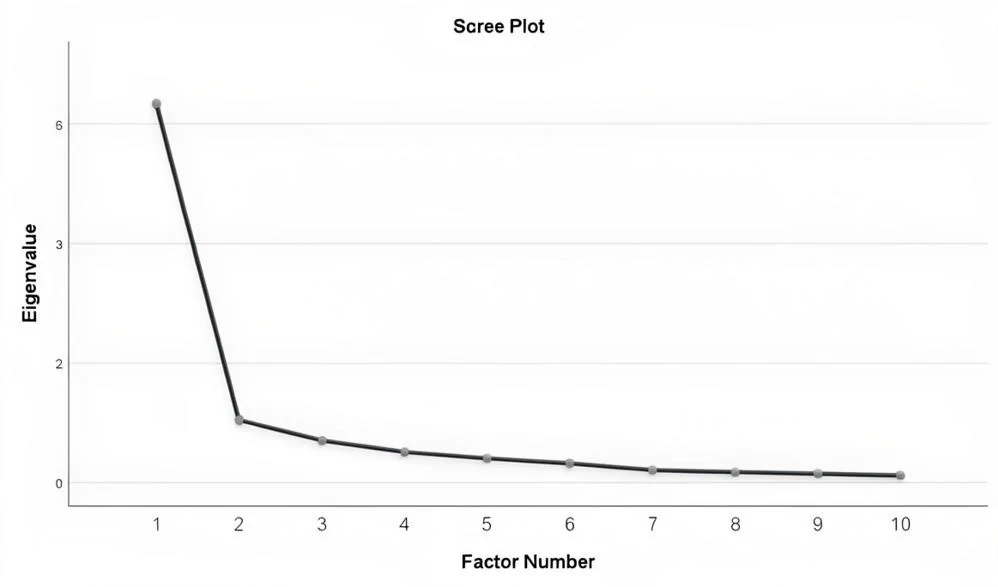
Scree Plot of Eigenvalues for the Persian Version of the HPSQ

 The results of the Kaiser-Meyer-Olkin test and Bartlett’s test of sphericity showed that the study sample was adequate (0.876) and that the correlation matrix was suitable for factor analysis (χ 2 = 144 and *P* < 0.001). While this ratio exceeds the more conservative < 3 threshold ^18^, the chi-square statistic is sensitive to sample size (n = 203). The goodness-of-fit indices for the CFA are presented in Table 4. The fit index values showed that this model fits well. The road map for the structure of the HPSQ is provided in Figure 2.

**Table 4:** Confirmatory Factor Analysis Error and Goodness of Fit Indices of Scale for the Persian version of HPSQ (n = 203)

**Fitness Criteria**	**Acceptable Fitness**	**Results**
χ 2 /Df/*P*	0 < χ 2 /sd < 3 - *P* < .05	144/34/*P* = 0.00
GFI	GFI > 0.90	0.91
AGFI	0.80 ≤ AGFI ≤ 0.90	0.81
RMR	RMR < 0.05	0.05
NFI	NFI > 0.90	0.93
NNFI	NNFI > 0.90	0.93
IFI	IFI > 0.90	0.95
CFI	CFI > 0.90	0.95
RMSEA	0.05 ≤ RMSEA ≤ 0.10	0.1

χ^2^/Df: Chi-Square/Degrees of Freedom, GFI: Goodness of Fit Index, AGFI: Adjusted Goodness of Fit Index, RMR: Root Mean Square Residual, NFI: Normed Fit Index, NNFI: Non-Normed Fit Index, IFI: Incremental Fit Index, CFI: Comparative Fit Index, and RMSEA: Root Mean Square Error of Approximation

**Figure 2:**
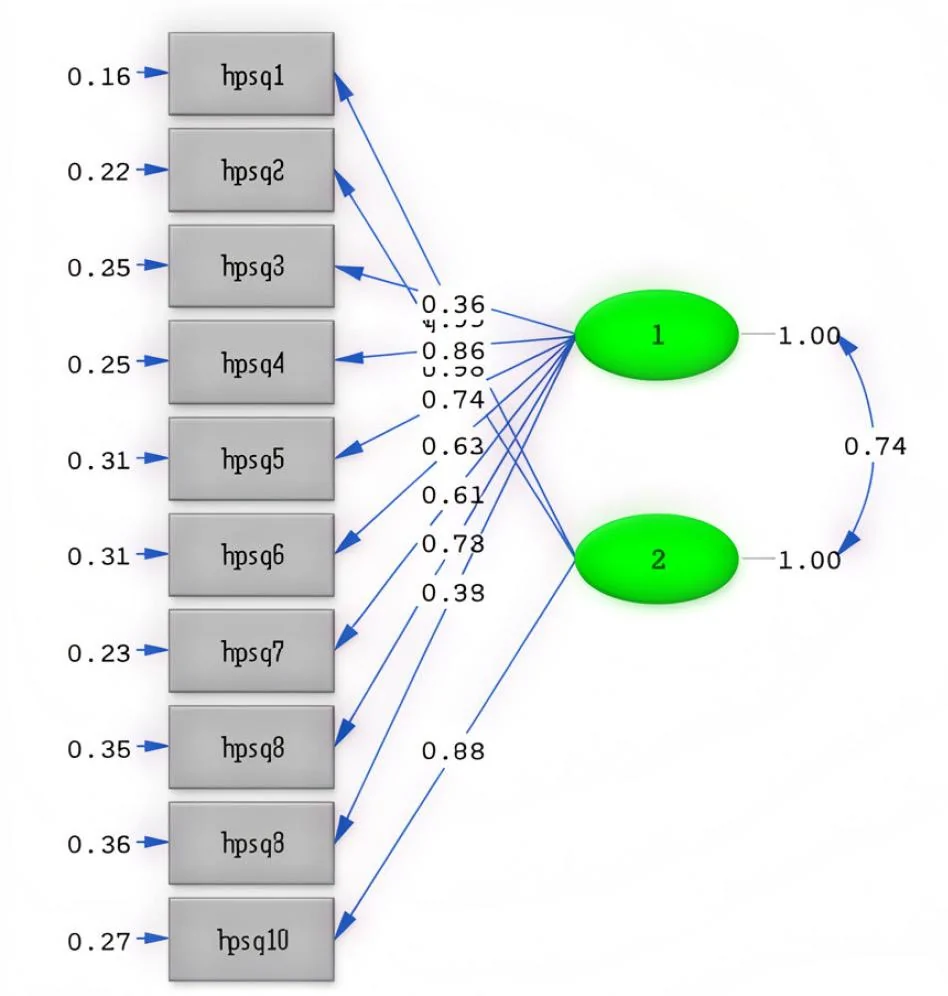
Significance Levels of the Rates, Latent Variables, and Explained Observed Variables for the Two-Dimensional Model of the Persian Version of HPSQ

 Cronbach’s α coefficient was 0.93, indicating excellent internal consistency ^19^. The ICC values ranged from 0.89 to 0.96 (n = 43), indicating excellent test-retest reliability of the test ^20^.

## Discussion

 This study evaluated the psychometric properties of the Persian version of the HPSQ. The results demonstrate that it is a valid and reliable tool for teachers to screen handwriting problems in Iranian second and third-grade students. Handwriting is a fundamental skill in educational activities at school. Problems related to handwriting can indicate learning disabilities in children.^21^

 The findings align with previous research, confirming that the HPSQ is a simple, clear, and effective screening questionnaire. This questionnaire is completed by teachers. However, a study has shown that in the second through fifth grades, there is a high correlation between the scores obtained from the teachers’ evaluation and the students’ own opinions.^15^ Therefore, the results of this questionnaire can also be used for client-centered goal-setting.

 The results of discriminant validity showed that the Persian HPSQ could distinguish between the handwriting skills of second and third grade students, which is consistent with other language versions that found significant differences in mean scores across grades.^3,4^ This is expected, as handwriting quality and fluency typically improve from one grade to the next.^22,23^ The present study found significant differences between grade 2 and grade 3 students across all HPSQ items (*P* < 0.05), with effect sizes ranging from 0.4 to 0.98. The medium to large effect sizes (d ≥ 0.50) observed for 6 items suggest that these improvements are practically meaningful. However, items with small effect sizes (d < 0.50) may indicate that certain aspects of handwriting develop more gradually or are less sensitive to grade differences. Future research should continue to examine the magnitude of these differences using effect size calculations.

 The CFA revealed an acceptable fit for the two-factor model. The RMSEA of 0.10 fell within the acceptable range (0.05 ≤ RMSEA ≤ 0.10) and the CFI of 0.95 exceeded the excellent threshold,^24^ supporting the structural validity of the model. The χ^2^/df ratio was 4.24, which falls below the commonly accepted cutoff of 5.^24^ While the RMSEA value is at the upper acceptable limit, the CFI and other fit indices collectively support the model’s adequacy. The AGFI was 0.81, which is borderline. However, AGFI is conservative and can underestimate fit in smaller samples (n = 203).

 Consistent with the original and Spanish versions, our EFA loaded the items onto two components. The results of the study were exactly the same as the HPSQ developer’s study and the Spanish version. The first component (items 3-9) relates to well-being and performance time, while the second (items 1, 2, and 10) pertains to legibility. It should be noted that confirmatory factor analysis was not performed in Rosenblum’s original study.^13,25^ Results of confirmatory factor analysis in the Thai and Czechian versions revealed that measurement items were loaded into three components (items 1, 2, and 10 (legibility); items 3, 4, and 9 (performance time); and items 5, 7, 6, (physical and emotional well-being)). In these studies, exploratory factor analysis was not used.^3,4^ The Thai validation included students in grades 1–4 (ages approximately 6–10 years), ^4^ and the Czech validation included students in grades 3–4 (ages approximately 8–10 years).^3^ In contrast, our study was restricted to grades 2 and 3 (ages 8–10 years). While our sample overlaps considerably with the Czech sample (grades 3–4) and partially overlaps with the Thai sample (grades 1–4), the inclusion of grade 1 students in the Thai validation may have introduced greater developmental diversity. Handwriting proficiency is a developmental skill that evolves rapidly across primary school years. Younger children (grades 1–2) are still mastering basic motor control and letter formation, while older children (grades 3–4) have developed more automated handwriting processes.^22^ Cultural and educational factors may also contribute to the observed discrepancy. The Persian script is a cursive, right-to-left writing system with distinct visual and motor demands compared to the scripts used in Thai and Czech writing systems. Translation and cultural adaptation may have influenced the factor structure. Although we followed standard forward-backward translation procedures,^26^ subtle differences in item interpretation across languages and cultures can affect how participants respond to specific items.

 All HPSQ items are designed to evaluate key aspects of handwriting and effectively identify related challenges. In other words, according to the results of this study, the internal consistency of this questionnaire is excellent.^17^ Previous studies that were in other languages also showed similar results and the internal consistency of the HPSQ has been reported from acceptable to excellent.^4,25^

 The present findings indicate that the Persian version of the HPSQ has good temporal stability, with test-retest reliability comparable to the original version.^13^ This suggests that Iranian teachers can confidently use the HPSQ to identify handwriting problems in students. However, we acknowledge several limitations. Concurrent validity was not examined in the present study. Future research should examine the correlation between the Persian HPSQ and established performance-based handwriting measures, such as the Evaluation Tool of Children’s Handwriting (ETCH) or the Minnesota Handwriting Assessment, to establish concurrent validity. While this study establishes discriminant validity based on grade-level sensitivity, future research should compare HPSQ scores between children with and without diagnosed handwriting difficulties to further strengthen the evidence for discriminant validity.

## Conclusion

 The Persian version of the HPSQ has acceptable validity and reliability for screening handwriting problems in Iranian school students in grades 2 and 3. It is a practical tool that can be used by teachers and professionals across various settings to identify children in need of further assessment and intervention.

## Competing Interests

 The authors report there are no competing interests to declare.

## Ethical Approval

 This study was approved by the Ethics Committee of Tabriz University of Medical Sciences (IR.TBZMED.REC.1403.127).”
